# Development of a Lateral Flow Highway: Ultra-Rapid Multitracking Immunosensor for Cardiac Markers

**DOI:** 10.3390/s19245494

**Published:** 2019-12-12

**Authors:** Nadezhda A. Byzova, Yuri Yu. Vengerov, Sergey G. Voloshchuk, Anatoly V. Zherdev, Boris B. Dzantiev

**Affiliations:** 1 A.N. Bach Institute of Biochemistry, Research Centre of Biotechnology of the Russian Academy of Sciences, Leninsky prospect 33, 119071 Moscow, Russia; nbyzova@inbi.ras.ru (N.A.B.); Yuriy.vengerov@synteco.ru (Y.Y.V.); zherdev@inbi.ras.ru (A.V.Z.); 2 Mental Health Research Center, Kashirskoye shosse 34, 115522 Moscow, Russia; vsg9036150341@yandex.ru

**Keywords:** immunochromatographic assay, multiassay, microfluidics, pin applicators, diagnostic tests, biomarkers

## Abstract

The integration of several controlled parameters within a single test system is experiencing increased demand. However, multiplexed test systems typically require complex manufacturing. Here, we describe a multiplexed immunochromatographic assay that incorporates a conventional nitrocellulose membrane, which is used together with microspot printing, to construct adjacent microfluidic “tracks” for multiplexed detection. The 1 mm distance between tracks allows for the detection of up to four different analytes. The following reagents are applied in separate zones: (a) gold nanoparticle conjugates with antibodies against each analyte, (b) other antibodies against each analyte, and (c) antispecies antibodies. The immersion of the test strip in the sample initiates the lateral flow, during which reagents of different specificities move along their tracks without track erosion or reagent mixing. An essential advantage of the proposed assay is its extreme rapidity (1–1.5 min compared with 10 min for common test strips). This assay format was applied to the detection of cardiac and inflammatory markers (myoglobin, D-dimer, and C-reactive protein) in human blood, and was characterized by high reproducibility (8%–15% coefficient of variation) with stored working ranges of conventional tests. The universal character of the proposed approach will facilitate its use for various analytes.

## 1. Introduction

The rapid progress in medical diagnostics, testing of consumer products, environmental monitoring, biosafety, and industrial control have established new requirements for the detection of biologically active compounds. To ensure prompt and informed decision making, detection methods should provide information regarding the presence and quantity of a significant number of compounds in a minimal amount of time [1]. This trend has led to the transfer of testing from specialized laboratories to point-of-care (POC) sites, with the development of analytical devices that do not require additional tools and reagents or laborious manipulations [2].

Immunochromatographic assays (also known as lateral flow assays or test strip assays) represent the most successful approach for POC diagnostics [3,4]. The devices for these assays (test strips) typically comprise multimembrane composites with preapplied reagents. Contact of the test strip with the sample leads to the movement of the sample solution along the membranes, and to the binding of a detectable label in certain zones of the strip [5]. The duration of the analysis is determined by the time it takes for the solution to move and for specific labeled complexes to form in sufficient quantities. This duration depends primarily on the viscosity of the sample and the choice of composite membranes used in the composite; typically, it is about 10–15 min [6]. Compared with other immunoanalytical methods, lateral flow assays reduce the time to result severalfold. However, in many cases (for example, when providing emergency medical care), further acceleration and increased performance are still in demand.

**Figure 1:**
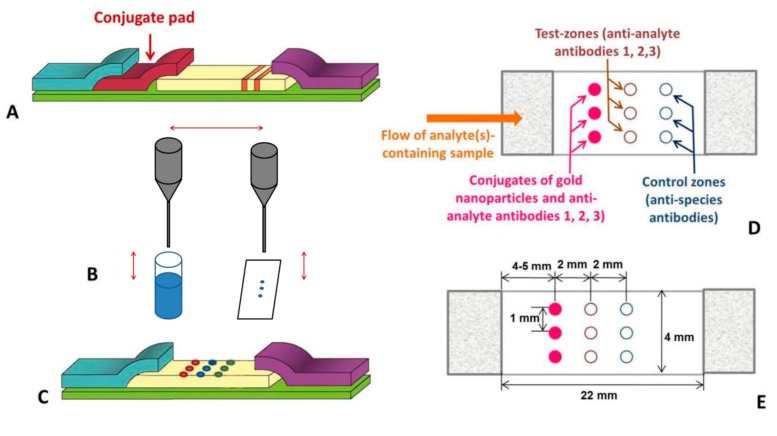
Immunochromatographic test strips in classic and proposed formats. (**A**) Classic format comprising a conjugate pad and lines of applied reactants at the working membrane. (**B**) Proposed format comprising the microdrop application of the conjugate onto the working membrane with an automated pin device. (**C**) Proposed format with an ordered array of points containing different reactants at the working membrane. (**D**) Geometry of the reactant application in the proposed format, where three biomarkers are being analyzed. (**E**) Distances between the applied reactants at the working membrane.

The classic format of immunochromatography does not provide such acceleration, as it involves the leaching of reagents (such as antibody–nanoparticle conjugates) from the pores of one membrane and their transition to another (Figure 1A). Here, we propose a rapid immunochromatographic assay where these antibody conjugates are applied not to a separate membrane, but to the edge of the working membrane in front of the zones for further binding. The conjugates are applied by mechanical contact between a moistened pin and the membrane (Figure 1B). As a result, the conjugate pad is excluded from the test system, along with the need for its impregnation, the dissolution of the conjugate, and the conjugate’s passage through the juncture of the pad and the working membrane.

Conventionally, multiplexing of the test strip is achieved by the sequential deposit of lines of reagents of different specificities on the working membrane (which increases the duration of analysis), or by combining independent test strips in one device (which increases the consumption of the materials and reagents) [7,8]. In the format proposed here, an ordered array of points on the membrane is used for the independent formation of several specific complexes (Figure 1C). The points for the initial location of the conjugate, the specific binding of the detected immune complexes, and the control binding of the conjugate are located adjacently to each other, 2 mm apart, along the length of the strip, with the tracks containing reagents of a different specificity spaced 1 mm apart (Figure 1D,E). Due to the differences in the properties of the liquids moving laterally along the tracks and between these points, the flows of specific reagents are not eroded and do not mix with each other. Thus, the standard geometry of the immunochromatographic test strip (4 mm wide) allows for the simultaneous testing of three or four analytes. In addition, with this format, a separate control point for each test analyte indicates the quality of the antibody conjugates toward their respective analyte. This is in contrast to common multiplexed test strips, where only one control line is present and is colored by reactants of different specificities. Moreover, the transition from the line to spot-application of immune reagents allows for nearly fivefold reductions in their consumption per assay.

This study presents the development and characterization of a test system that implements the proposed approach for the ultra-rapid and simultaneous determination of three diagnostically important biomarkers: myoglobin (Myo), C-reactive protein (CRP), and D-dimer (DDm). These biomarkers are usually assayed after myocardial infarction to confirm the diagnosis and to forecast further risks as well as to assess the severity of the inflammatory processes accompanying the incident [9].

## 2. Materials and Methods

### 2.1. Reagents and Materials

Human Myo, CRP, and DDm; monoclonal anti-Myo antibodies; the Myo-M7 clone; monoclonal anti-CRP antibodies; the CRP-5E9 and CRP-4C6 clones; monoclonal anti-DDm antibodies; and the DDm-41D2 and DDm-25D5 clones were purchased from Bialexa (Moscow, Russia). Another Myo-A6 clone was obtained from the Russian Research Centre of Molecular Diagnostics and Therapy (Moscow, Russia). Goat anti-mice immunoglobulin antibodies (GAMI) were obtained from Arista Biologicals (Allentown, PA, USA). Tris, sucrose, sodium citrate, sodium azide, and chloroauric acid were obtained from Sigma-Aldrich (St. Louis, MO, USA), and the bovine serum albumin (BSA) was from Biowest (Nuaille, France).

Solutions for the synthesis of the gold nanoparticles (GNPs) and their conjugates, with various antibodies, were prepared using deionized water; the resistivity of which was no less than 18.2 MΩ cm (Simplicity System; Millipore, Bedford, MA, USA).

To manufacture the test strips, a nitrocellulose membrane grade CNPC with a pore size of 15 μm, fixed blood separation membrane FR-1, and absorption membrane AP045 (all from Advanced Microdevices, Ambala Cantonment, India) were attached to the solid support.

### 2.2. Synthesis of Gold Nanoparticles and Their Antibody Conjugates

GNPs with a 30-nm average diameter and their antibody conjugates were prepared as previously described [10]. Briefly, 1.0 mL of a 1% (*w*/*v*) aqueous solution of chloroauric acid was added to 97.5 mL of water. The mixture was heated under reflux, and 1.5 mL of 1% (*w*/*v*) sodium citrate solution was added. After refluxing for 30 min, the GNP preparation was cooled and stored at 4 °C. For the antibody conjugation, the obtained GNP solution (OD_520_ = 1.0; pH 9.0) was added to Myo-A6, CRP-5E9, or DDm-41D2 antibody solutions at concentrations of 8, 14, and 8 μg/mL, respectively. The mixtures were incubated for 30 min at 20–22 °C under stirring, after which an aqueous BSA solution was added to a final concentration of 0.25% (*w*/*v*). GNPs with immobilized antibodies were separated from nonreacted antibodies by centrifugation at 10,000× *g* for 30 min. After the supernatant was removed, the residue was resuspended in a buffer comprising 0.02 M Tris-HCl, pH 7.6; 6.0% BSA; 12% sucrose; and 0.1% sodium azide (TBSS; all *w*/*v*) for storage at 4 °C.

### 2.3. Test Strip Manufacturing

The microarrays were spotted by touching the working membrane with a steel pin prewetted with the conjugate, as previously described [11]. The total length of the CNPC membrane in the constructed multimembrane composite was 26 mm, and the regions 2 mm from each end were covered by the auxiliary membranes. The GNP–antibody conjugates were applied 6 mm from the edge of the working membrane, using solutions with OD_520_ = 15 in TBSS. Unlabeled antibodies Myo-M7, CRP-4C6, and DDm-25D5 were applied 2 mm away from the conjugate zone using solutions with concentrations of 2 mg/mL in PBS containing 0.1% BSA, 0.1% sucrose, and 0.1% sodium azide (all *w*/*v*). GAMI were applied 2 mm away from the antibody zone using a solution with a concentration of 2 mg/mL, in the same buffer as that used for the unlabeled antibodies. After the immunoreagent application, the CNPC membrane was dried in air at 20–22 °C for at least 20 h. The membranes were then assembled and cut into test strips 4.0 mm in width.

### 2.4. Immunochromatographic Assay

The assay was performed at room temperature. Blood serum (60 μL) was applied to the blood separation membrane of the test strips. The movement of the sample and the formation of the stained spots were complete after 3 min. The binding of the labeled reagents in the control and test zones was recorded with a CanoScan 9000F scanner (Canon, Tokyo, Japan), followed by image processing using TotalLab 2.1 software (TotalLab, Newcastle upon Tyne, UK), as described in the literature [11].

## 3. Results

Our choice of immune reactants and the method of their conjugation were based on our previous developments of test strips for these analytes using the traditional format [10,12]. The selected mode of spot-applications on these membranes leads to the formation of colored spots (both initially applied and formed in the test and control zones) with a diameter of 0.6–0.7 mm. Such sizes are optimal for the proposed analysis. The achieved distance between spots on adjacent tracks of 0.2–0.3 mm or more excludes mixing of flows for reagents of different specificity. When this distance is reduced, cases of closure for adjacent spots appear. Reducing the spots requires a change in the size of the pin and increased time of application for the same volume. This reduction also increases the variation in the quantitative results when testing replicates of the same sample.

**Figure 2:**
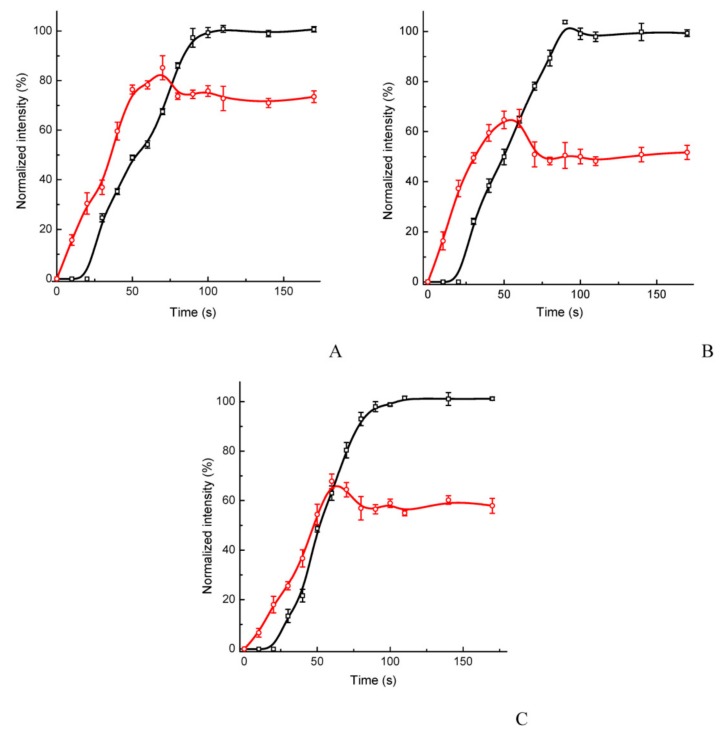
Kinetics of the coloration of the test (red lines) and control (black lines) zones of the multitrack immunochromatographic assay. The tested blood serum samples contained 0.3 μg/mL of Myo (**A**), 3 μg/mL of CRP (**B**), and 3 μg/mL of DDm (**C**). Values represent the mean ± standard error of the mean (SEM; n = 4).

The multitrack assay demonstrated a high rapidity; as for all the analytes, a saturation of the test zone was reached within 50–60 s after initiating the assay, and the control zone reached saturation within 80–90 s (Figure 2). The dynamics of the reactants’ movement and binding is also presented in the Supplemental Videos S1–S3.

**Figure 3:**
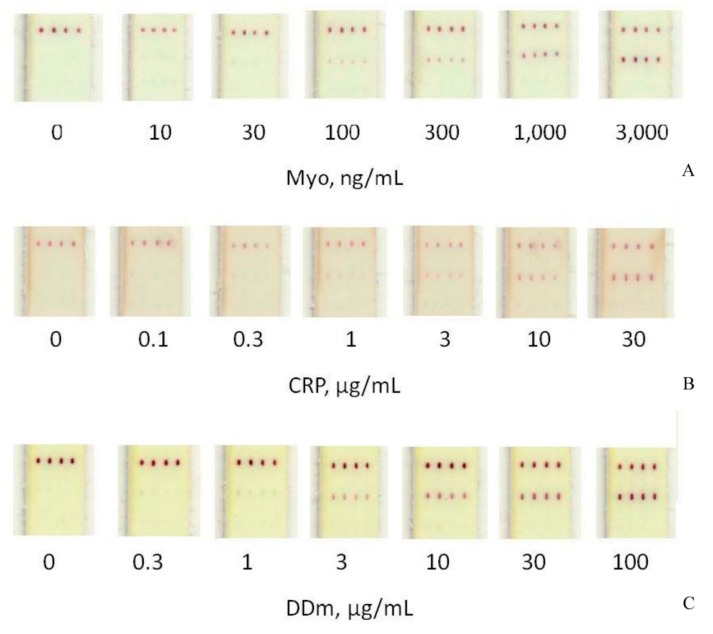
Multitrack immunochromatographic assay of myoglobin (**A**), C-reactive protein (**B**), and D-dimer (**C**) in four repeated tracks. Appearance of the working membrane after the completion of the assay.

The assay of the serum samples was realized without any nonspecific coloration of the working membrane, even at high concentrations of the analytes (Figure 3).

**Figure 4:**
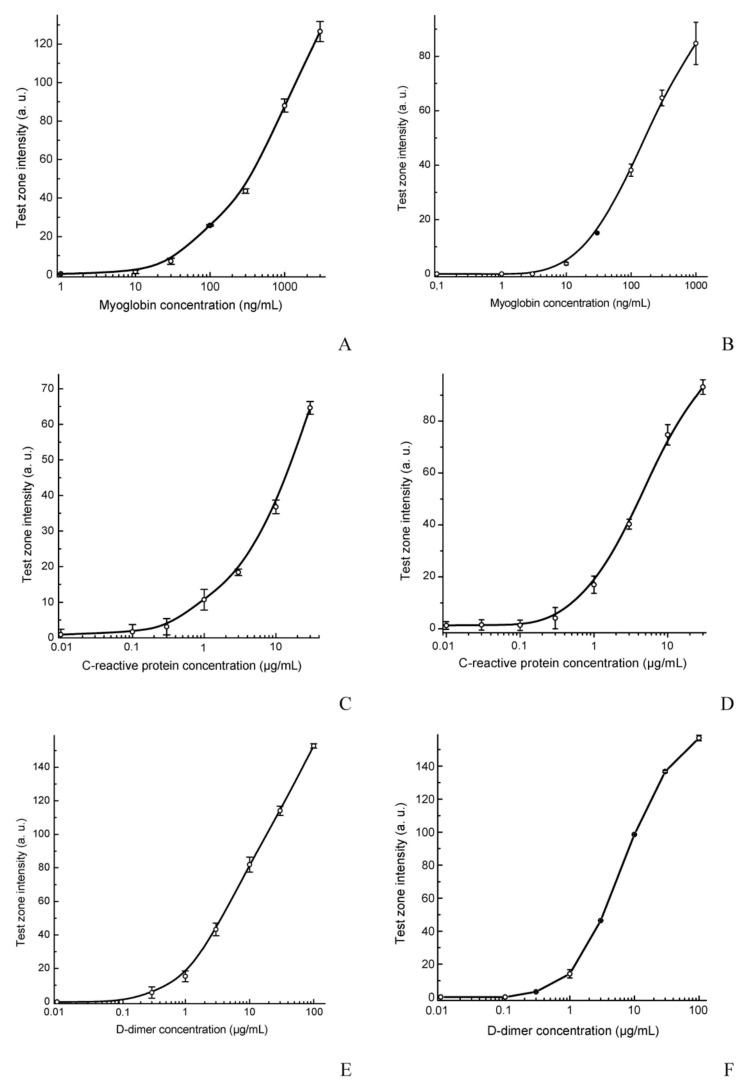
Calibration curves of the multitrack (**A**,**C**,**E**) and common test strips for the singleplexed detection (**B**,**D**,**F**) of myoglobin (**A**,**B**), C-reactive protein (**C**,**D**), and D-dimer (**E**,**F**). Values represent the mean ± SEM (*n* = 4).

The concentration dependences indicated the working ranges of the assays to be similar to that of common traditional tests (Figure 4). Using our assay, the minimal detectable concentrations were 0.03 μg/mL for Myo and 0.3 μg/mL for CRP and DDm. Despite the reduced area of the binding zones, the photometric assay was characterized by good reproducibility, as follows: samples with 0.1 μg/mL of Myo, 3 μg/mL of CRP, and 1 μg/mL of DDm had coefficients of variation (*n* = 4) ranging from 8% to 15%.

**Figure 5:**
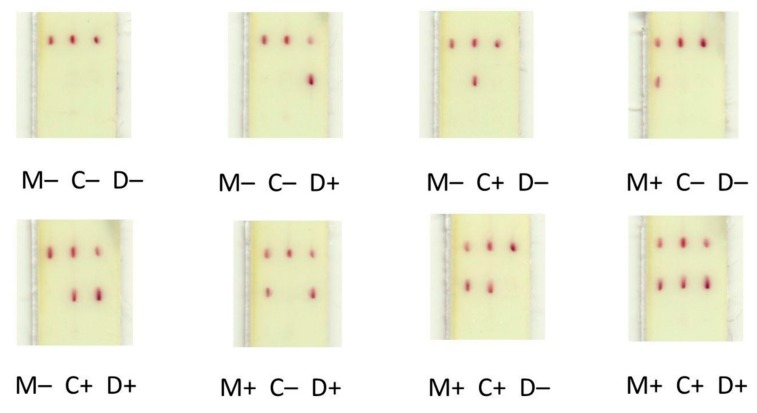
Appearance of the strip test and control zones following the assay of the serum samples containing different combinations of analytes (described below the strip images). M−, no Myo; M+, 3 μg/mL Myo; C−, no CRP; C+, 30 μg/mL CRP; D−, no DDm; D+, 30 μg/mL DDm.

**Figure 6:**
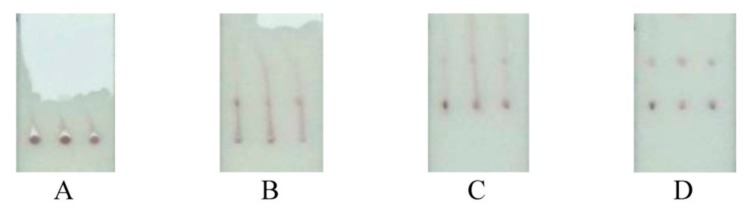
Sequential images (**A**–**D**) of a test strip during the movement of the gold nanoparticle conjugates along the membrane and its binding within the test and control zones. The sample contains 3 μg/mL of Myo, 30 μg/mL of CRP, and 30 μg/mL of DDm.

No cross-reactivity was observed between the primary antibodies and the analytes (Figure 5). This was due to the focused flow of the reactants along individual tracks between the conjugate, test, and control zones (Figure 6), which was facilitated by the differing composition and viscosity of the solutions within and between the tracks.

To characterize stability of the prepared test strips, they were stored in sealed aluminum bags with silica gel as a desiccant. It was found that storage for 4 months at room temperature did not cause reliable changes in values of GNPs binding for any of the three analytes, nor did it lead to nonspecific coloration for testing the samples without the analytes.

## 4. Discussion

With the application of all reagents to the working membrane, the simplification of the test strip significantly reduced the analysis time. This proposed approach excludes the requirement for the gold conjugate solution to dissolve at the conjugate pad and then to transfer from one membrane to another with the accompanying longer duration of lateral flow. In the proposed format, the detected complexes are formed after the sample has rapidly moved along the tracks on the working membrane between neighboring zones, which are only 2 mm apart. Several immunochromatographic tests that exclude the conjugate pad have been reported [13,14,15]. In these assays, the conjugate is preincubated with the sample beyond the test strip. However, in such tests, the distance that the conjugate needs to move has not been reduced, and the preincubation step further increases the analysis time.

The point application of reagents permits the simultaneous determination of several analytes with a low consumption of reagents and materials. In earlier developments of immunochromatographic tests with the point application of reactants [11,16], the separation of the binding zones of different specificities was not accompanied by the separation of conjugates of different specificities. As a result, during the movement of the liquid front, the majority of conjugates passed outside the binding zone and were, therefore, lost. Furthermore, the previously described integration of the parallel flows of reagents of different specificities was accomplished with a significant increase in the complexity of the test strip, through additional modifications of the working membrane or the inclusion of additional components in the test strip [17,18,19,20].

The cause of the focused flow of the reactants along individual tracks is the laminar motion of the fluid along the working membrane. It was shown earlier [21] that the Reynolds number for typical immunochromatographic membrane is two to four orders of magnitude lower as compared with its critical value for laminar flow in a porous medium. Under these conditions, flows of nearby liquids that differ in composition do not mix with each other during their lateral flow movement [22]. Accordingly, the conjugate of GNPs—washed out from the point of its initial application on the working membrane—consistently reaches the sites of its specific binding in the test and control zones.

Due to this focused flow of the reactants, the manufacture of multiplex systems is simplified in comparison with traditional multiplex microfluidic tests, in which flows with reagents of different specificity are separated by additionally formed impermeable barriers.

The proposed assay principle is universally applicable; it may be transferred for immunodetection of other analytes with different variants of immune complex formation (sandwich assays, competitive assays, etc.). The main demand for sample matrices is the stored nonmixed movement of the tracks. Immunoassays of hydrophobic compounds, such as mycotoxins and dioxins, include the use of water-organic extracting mixtures and so may need additional treatments of the working membrane.

Work with other matrices will require replacing the blood separation membrane with the other membrane carriers selected for these matrices when developing conventional lateral flow tests. The blocking reactants (such as BSA) could be moved, if necessary, to these starting membranes.

Although the risk of sensitivity loss for low-affinity antibodies should be controlled for the proposed rapid assay format, this does not constitute an obstacle to the application of the proposed format. Achieving equilibrium in 1–2 min for solutions with a high concentration of labeled antibodies (necessary for their efficient detection) was demonstrated in a number of homogeneous immunoassay formats [1].

In the proposed analysis format, the slow-release of the GNPs conjugate from the additional membrane is excluded. The conjugate, initially localized on a limited area of the working membrane, not only reaches the binding zones quickly, but also quickly leaves them in the absence of interaction.

Another significant advantage of the proposed method is the objectivity of assessing the quality of reagents by the control zone, because each point here indicates the quality of the conjugate of the antibodies against a specific analyte. When using common multiplex test strips, the coloration of the single control zone is caused by reactants of different specificities and does not provide information about the antibodies to each analyte.

The format of the immunochromatography implemented in the present study provides both a reduction in the analysis time (1 min for coloration of the test zone and 1.5 min for that of the control zone) and a more efficient multiplexed analysis. These improvements do not require the introduction of additional components or complications in the manufacturing process.

The proposed analysis versus other compact multiplex analytical systems [23] offers the following advantages:

Compared to immunochromatography using combined individual test strips—faster assay and reduction in the consumption of reagents and materials;
Compared to immunochromatography using sequentially located test zones—faster assay and reduction in the consumption of reagents;
Compared to paper microfluidic devices—simple manufacturing and simple control of the reactions sequence;
Compared to biochips—formation of detectable complexes upon flow through a porous carrier instead of diffusion-dependent binding to the substrate.

Upper limit of simultaneously detected compounds for the proposed assay format varies from 5–6 (standard width of test strips) to 10–20 (cutting to strips with increased width).

## 5. Conclusions

The experimental performance of the proposed assay format demonstrated its rapidity and effectiveness. Using this assay, three analytes could be simultaneously detected on the same test strip, with a reduction in the analysis time to 1–1.5 min. The consumption of specific reagents (per assay of one analyte) was reduced up to fivefold without any loss in sensitivity. The applicability of the proposed format for testing a complex biomatrix, such as blood serum, was shown. The universal nature of this approach allows for its use in various clinically and otherwise significant analytes.

## Supplementary Materials

The following are available online at https://www.mdpi.com/1424-8220/19/24/5494/s1, Supplementary Video 1: testing sample with 0 µg/mL of myoglobin, 0 µg/mL of C-reactive protein, and 0 µg/mL of D-dimer. Supplementary Video 2: testing sample with 0.3 µg/mL of myoglobin, 3 µg/mL of C-reactive protein, and 3 µg/mL of D-dimer. Supplementary Video 3: testing sample with 3 µg/mL of myoglobin, 30 µg/mL of C-reactive protein, and 30 µg/mL of D-dimer.
