# Supplementary material for: Development of a Lateral Flow Highway: Ultra-Rapid Multitracking Immunosensor for Cardiac Markers
Source: Sensors (Basel). 2019 Dec 12;19(24):5494. doi: 10.3390/s19245494 (PMC6970229; doi:10.3390/s19245494)
Supplement: Supplementary file 1 [file sensors-19-05494-s001.zip › Supplements/English-editing-certificate-Boris Dzantiev.pdf]

We certify that the following article

Lateral flow highway: Ultra-rapid multi-tracking immunosensor for cardiac markers

Boris Dzantiev

has undergone English language editing by MDPI. The text has been checked for correct use of grammar and common technical terms, and edited to a level suitable for reporting research in a scholarly journal.
MDPI uses experienced, native English speaking editors. Full details of the editing service can be found at
► https://www.mdpi.com/authors/english.

Multidisciplinary Digital Publishing Institute

Basel, November 2019

Martyn Rittman, Ph.D.
English Editing Manager
englishediting@mdpi.com
https://www.mdpi.com/authors/english
